# Dual task interference during gait in patients with unilateral vestibular disorders

**DOI:** 10.1186/1743-0003-7-47

**Published:** 2010-09-20

**Authors:** Alberto Nascimbeni, Andrea Gaffuri, Arminio Penno, Mara Tavoni

**Affiliations:** 1 Rehabilitation Unit, S. Croce Hospital, Moncalieri Turin, Italy; 2 E.N.T. Unit, S. Croce Hospital, Moncalieri Turin, Italy; 3 Rehabilitation Unit, Martini Hospital, Turin, Italy

## Abstract

**Background:**

Vestibular patients show slower and unsteady gait; they have also been shown to need greater cognitive resources when carrying out balance and cognitive dual tasks (DT). This study investigated DT interference during gait in a middle-aged group of subjects with dizziness and unsteadiness after unilateral vestibular neuronitis and in a healthy control group.

**Methods:**

Fourteen individuals with subacute unilateral vestibular impairment after neuronitis and seventeen healthy subjects performed gait and cognitive tasks in single and DT conditions. A statistical gait analysis system was used and spatio-temporal parameters were considered. The cognitive task, consisting of backward counting by three, was tape recorded and the number of right figures was then calculated.

**Results:**

Both patients and controls showed a more conservative gait during DT and between groups significant differences were not found. A significant decrease in cognitive performance during DT was found only in the vestibular group.

**Conclusions:**

Results suggest that less attentional resources are available during gait in vestibular patients compared to controls, and that a priority is given in keeping up the motor task to the detriment of a decrease of the cognitive performance during DT.

## Background

Vestibular information is important during gait together with vision and somatosensory input [1]. In fact, galvanic vestibular stimulation, experimentally producing functional imbalance between the two vestibular apparatus, causes in healthy subjects gait deviation to the anodal side [2]. Moreover, gait unsteadiness is a common complaint after vestibular neuritis and a higher fall risk has been also demonstrated in these patients [3] who often need to undertake a vestibular rehabilitation program in order to promote functional recovery.

Previous studies have shown in these patients spatio-temporal gait changes, disruption of head-trunk coordination and decrease of head movements [4-9]. Furthermore, the presence of a cognitive-vestibular interaction and the complaint of poor concentration, memory impairment [10] and disrupted navigation [11] has been also highlighted in vestibular patients.

The dual task (DT) paradigm, by simultaneously employing balance and cognitive tasks, has been used in recent years to shed light on motor and cognitive interference [12] in healthy young people [13,14], elderly subjects [15] and patients. In particular, cognitive and motor interference during gait has been studied in pathologies such as stroke [16], Parkinson disease [17] and dementia [18]. These studies are based on the assumption that cognitive resources are limited and can be undermined by the execution of concurrent tasks, especially when pathologies limit the performance by a reduction of attentional resources available or by the need of increased attention to carry out usual tasks owing to motor-sensory impairments. Moreover, the degree of impairment of motor-sensory integration during DT will depend on the amount of attentional resources required by the proposed tasks [14,19].

Motor-cognitive interference has been previously demonstrated during standing balance tasks in vestibular patients. In fact, vestibular impairments require increased attention for postural and balance control also in a compensated phase. However to our knowledge, the possible DT interference during a common but more attention requiring condition such as gait, has never been studied before in vestibular patients. This issue is of particular interest in the subacute phase, when vestibular rehabilitation programs are usually carried out to improve dynamic balance which in the activities of daily living are commonly challenged by DT.

The main aim of this study was to explore DT interference in a homogeneous group of patients with unilateral vestibular impairment while performing backward counting during gait, comparing the performances during single and DT. A second aim was to find out differences if any with an healthy control group. Since gait and balance impairments increase with age, together with the need of attentional resources, causing more DT interferences [12], older adults were not recruited in this study.

## Methods

### Participants

Patients and control subjects were included if their age was between 18 and 65 years and without other pathologies interfering with gait, balance and cognition. 14 patients with unilateral vestibular impairment after vestibular neuritis and 17 healthy control subjects were evaluated. Mean age, height and weight of the patient group, made up of 8 female and 6 male, were 45.2 ± 8.1 years, 169 ± 9.8 cm and 66.4 ± 13.4 Kg while the values of the control group, made up of 13 female and 4 male, were 44.1 ± 7.9 years, 167 ± 10 cm. and 69.8 ± 14.3 Kg respectively. The two groups did not result statistically different for the aforementioned parameters. All patients enrolled were referred for vestibular rehabilitation for enduring complaints of dizziness or unbalance caused by the previous neuritis. The mean time elapsed from the acute phase was 5.1 ± 6.2 months (range 1-24) and nervous system suppressant medications were no more used at the time of the assessment. The patients were first assessed by a neurotologist and underwent alternating binaural bithermal caloric stimulation, head positioning test and audiometry. Vestibular tests showed 7 patients with right and 3 with left hypofunction, 2 with right and 2 with left loss of vestibular function. Brain MRI was also carried out in order to rule out other vestibular or central nervous system pathologies that can affect balance such as cerebello-pontine angle or white matter diseases. As these tests did not demonstrate any other pathologies, the diagnosis of peripheral unilateral vestibular impairment was confirmed.

All patients filled in a Dizziness Handicap Inventory (DHI) questionnaire for disability assessment (median 50, range 20-80).

All subjects signed a written informed consent and the study was approved by the Orbassano A.S.O. San Luigi Gonzaga Ethical Committee, conforming with Geneva convention.

### Tasks and procedures

The motor single task consisted of walking at self selected speed, back and forth in a well-lit gait laboratory without stopping. The distance between each turn was of 12 m. The cognitive single task consisted of backward counting aloud by 3 while the subjects were comfortably seated. During DT participants were asked to walk while backward counting without any prioritization of cognitive or motor task, and to carry out the test to the best of their abilities. In case of miscalculations, they were instructed to continue counting from the last spoken digit.

Each participant performed three trials. Before the beginning of the test, participants carried out a walking practice trial of about 1 minute wearing gait analysis sole sensors and performed a brief counting practice, showing to have understood the task. The order of the trials was randomised and each trial lasted one minute. Each participant underwent all test conditions during the same day.

STEP 32 gait analysis system (DEM Italia, Leinì, Turin, Italy) was employed for gait assessment. Phases of contact of the feet with the ground on a three level scale (heel, sole and forefoot) were acquired from three adhesive footswitches placed under the first and fifth metatarsal head and under the posterior part of the heel of each bare foot. The acquired data were offline statistically processed by the system software. The sampling rate was of 2 KHz and footswitches closing strength was of 3 N. The statistical gait analysis system employed allows atypical gait patterns such as those recorded during turns and acceleration-deceleration phases to be excluded from analysis. The parameters evaluated were: foot contact (FC), defined as the stride phase percentage in which all three foot switches were in contact with the ground, swing (Sw) and double support (DS) percentages of stride, stride time (ST) and coefficient of variance of stride time (CV). CV was calculated as the percentage of the quotient between standard deviation and stride time mean (CV = [SD/ST mean] × 100). For each gait parameter the mean value of right and left sides was calculated and used for statistical analysis.

The cognitive task consisted of backward counting by three, starting from 300. Previous studies demonstrated this cognitive task as sufficiently challenging in DT even in standing condition [14]. Single cognitive task and DT were tape recorded and the number of correct calculations were considered for further analysis.

### Data analysis

Mean values and standard deviations (SD) were used for gait parameters and cognitive performance. A paired t-test was used to assess gait and cognitive parameters changes between single and DT conditions within groups. An independent t-test was employed for between groups gait parameters comparisons in single and DT conditions. Owing to small sample groups separate t-test were chosen instead of ANOVA analysis and between groups analysis for the cognitive variable was not carried out for the same reason.

Normality of data distribution was verified with curtosis and skewness tests. When data distribution was not normal, Wilcoxon or Mann Whitney test were used accordingly. A value of *p *< 0.05 was deemed significant. Considering that 22 statistical tests were carried out, a Bonferroni correction was made to compensate for alpha inflation and a test-wise *p *< 0.002 was then accepted. The statistics were performed using Statgraphics Centurion software, release XV.

## Results

All patients and controls carried out the tasks properly. The analysis of gait parameters (Table 1) showed a significant increase of FC (patients *p *< 0.0005, controls *p *< 0.002), DS (patients *p *< 0.0001, controls *p *< 0.0007) and a significant decrease of Sw (patients *p *< 0.0005, controls *p *< 0.001) from single task to DT in both groups. Even though patients had higher mean values of FC, DS, ST and CV and lower mean values of Sw in both single and DT conditions than controls, these differences did not result significant when between-group comparisons were performed.

**Table 1:** Gait parameters values in each task by group, mean ± standard deviation.

Variables	Patients		Controls	
	**Single task**	**Dual task**	**Single task**	**Dual task**

**Foot contact (%)**	37.85 ± 3.91	40.9 ± 4.47*	36.02 ± 5.56	37.77 ± 5.33*
**Double support (%)**	22.85 ± 4.59	26.28 ± 4.48*	23.06 ± 4.51	24.42 ± 4.66*
**Stride time (S.)**	1.16 ± 0.12	1.22 ± 0.17	1.12 ± 0.10	1.16 ± 0.08
**Coefficient of variation of stride time (%)**	3.07 ± 1.77	3.25 ± 0.87	2.42 ± 0.83	2.93 ± 1.03
**Swing (%)**	38.51 ± 2.30	36.73 ± 2.20*	38.42 ± 2.24	37.65 ± 2.38*

S.: seconds; *significant difference (p ≤ 0.002) between single and dual task within group.

CV: Coefficient of variation = [standard deviation/mean] × 100.

For both groups, mean values of number of correctly enumerated figures during the cognitive task were higher under single task (patients: 22.85 ± 10.90, controls: 22.47 ± 9.28) than DT (patients:17 ± 9.33, controls: 19.76 ± 9.04). The total number of enumerated figures was also higher under single task (patients: 23.64 ± 10,53, controls: 23,71 ± 8,7) than DT (patients: 18.57 ± 8.83, controls: 21.18 ± 8.11). The within group differences between single and DT were significant for the patients group (*p *< 0.001) but not for controls (*p *= 0.034).

## Discussion

This study investigated cognitive and motor interference during gait in vestibular patients complaining of persistent dizziness and unsteadiness after unilateral vestibular neuritis. We decided to exclude from the study elderly subjects in order to rule out the effects of aging as previously suggested [20]. The main finding was that patients demonstrated a significant worsening of backward counting during gait, compared to backward counting in single task (carried out while seating), whereas the control group showed only a non significant trend in this direction. In regard to the gait task, both groups had a more conservative gait during DT. Moreover, the vestibular group showed a trend of higher mean values for FC, DS, ST and CV and lower values for Sw in both single and DT conditions than controls, even if between groups differences were not statistically significant.

The decreased cognitive performance of patients in DT suggests that more attentional resources are needed to cope with an unbalanced vestibular input during gait, causing motor-sensory integration disruption. In a condition of perceptual difficulty and cognitive overload, available attention is prioritized towards the gait task which is not significantly hindered, to the detriment of the cognitive task. In both groups the finding of significant gait changes of FC, DS and Sw between single and DT indicates the use of a more conservative gait when a demanding cognitive task is overloading attentional resources. This behaviour does not result to specifically involve vestibular patients, but it is also present in healthy participants even if with borderline statistical significance.

Our findings partially agree with previous gait analysis studies that demonstrated a disrupted gait pattern in vestibular patients. Greater foot pressure on the side of the lesion has been found in unilateral vestibular patients during gait with eyes closed [4]. Vestibular subjects were found to have increased ST and DS, lower gait speed and cadence [6,21] and higher interfoot distance during paced gait but only at increased speed [22]. The use of a trunk strategy for head stabilization, in order to compensate for a disrupted vestibulo-ocular reflex [5,7,8], and a decrease of head rotations while walking in dark was also demonstrated [9]. However, in our study, the between-groups gait differences were not significant, but only a trend was observed. This finding might be related to the type of our sample that included only middle-aged patients with unilateral vestibular impairment in a post-acute phase, allowing to rule out aging effect and acute phase related major balance disruption. Moreover, the gait analysis system used recorded and statistically analysed longer periods of walking, allowing a better representation of the more consistent individual gait pattern. Furthermore, CV of ST was previously found to be increased in healthy young subjects during DT [23] and greater SD of ST was also demonstrated in vestibular patients during gait [24]. However, we did not find any significant differences of CV: this observation might be explained by an inter-subjects variability in gait behaviour in the vestibular group as demonstrated by higher SD of CV in single task.

The priority of a postural strategy has been previously highlighted in a healthy young population during a standing and visual spatial memory DT [13]. On the other hand, a backward counting task was found to be a challenging task more than digit reversal and 2-bit classification tasks, increasing sway in healthy subjects standing on a compliant surface [14].

Previous studies on DT in vestibular patients, carried out with different balance and mental tasks and sometimes including peripheral and central balance impairments, had not produced unambiguous results. However, in agreement with our results, a decreased mental performance has been often evidenced and explained by a balance prioritization strategy. Longer reaction times were found in patients with vestibular balance disorders and controls in DT, with proportionately longer times while executing more difficult balancing tasks [19]. Both well compensated patients with unilateral vestibular loss and controls showed increased sway in DT but only patients had increased reaction times [25]. Other authors did not find out any impairment of a silent backward counting task during standing in a middle-aged sample of patients with central and peripheral balance dysfunction, but rather a decrease of sway, suggesting balance prioritization [20]. The performance of a visuospatial task deteriorated in both normal and vestibular patients while performing a balance task. However, a decrement in the cognitive performance and increased sway during computerized dynamic posturography was shown in patients with minor balance impairment and controls, but less sway was found during DT in patients with major balance problems [26], perhaps because of enhanced arousal.

## Conclusion

This study is in agreement with previous works suggesting a balance prioritization during DT in vestibular patients with a decrease of mental task performance. This behaviour might be the consequence of higher cognitive demands required to cope with unilateral vestibular impairment when multisensory integration and gaze stabilization [27] is needed during motor tasks. For the first time, our results extend this hypothesis to a more demanding and daily motor task such as gait, requiring greater multiple sensory integration. However, even if statistically significant, the differences of cognitive performance between the vestibular and the control groups are small and the clinical relevance of the results should be considered with caution.

Our findings, if confirmed in a greater sample, might be useful when planning DT dynamic exercises in order to improve DT performance in not well compensated vestibular patients. Further research could be useful to develop new strategies for rehabilitation and prevention of falls through tailored training in dual task exercises.

## Competing interests

The authors declare that they have no competing interests.

## Authors' contributions

AN was responsible for conception and design of the study, data analysis, and article drafting. AP clinically assessed and included participants, participating in the data discussion. MT and AG participated in data collection and article drafting. All authors read and approved the final manuscript.
